# Digital Phenotyping of Sensation Seeking: A Machine Learning Approach Using Gait Analysis

**DOI:** 10.3390/bs15091222

**Published:** 2025-09-09

**Authors:** Ang Li, Keyu Yang

**Affiliations:** Department of Psychology, Beijing Forestry University, Beijing 100083, China; yangkeyu@bjfu.edu.cn

**Keywords:** sensation seeking, gait, machine learning, digital phenotyping, OpenPose

## Abstract

Sensation seeking represents a significant risk factor for various mental health disorders and maladaptive behaviors, highlighting the need for objective assessment methods that circumvent the limitations of traditional self-report measures. This study introduces an innovative digital phenotyping approach that combines computational gait analysis with machine learning (ML) to quantify sensation-seeking traits and examines its validity. Natural gait sequences (using a Sony camera at 25 FPS) and self-report measures (Brief Sensation-Seeking Scale for Chinese, BSSS-C) were collected from 233 healthy adults. Computer vision processing through OpenPose extracted 25 skeletal keypoints, which were subsequently transformed into a hip-centered coordinate system and denoised using Gaussian filtering. From these kinematic data, 300 temporospatial gait features capturing various aspects of movement dynamics were derived. Using a supervised ML approach with feature selection, three ML models (SMO Regression, Multilayer Perceptron, and Bagging) were developed and compared through 10-fold cross-validation. The SMO Regression model demonstrated superior performance (*r* = 0.60, MAE = 3.50, RMSE = 4.59, R^2^ = 0.26), outperforming the other approaches. These results establish proof-of-concept for gait-based digital phenotyping of sensation seeking, offering a scalable, objective assessment paradigm with potential applications in clinical screening and behavioral research. The methodological framework presented here advances the field of behavioral biometrics by demonstrating how computer vision and ML can transform basic movement patterns into meaningful psychological indicators.

## 1. Introduction

Sensation seeking, defined by Zuckerman (1994) as “the seeking of varied, novel, complex, and intense sensations and experiences, and the willingness to take physical, social, legal, and financial risks for the sake of such experience”, has been consistently linked to mental health problems (e.g., substance use) and maladaptive behaviors (e.g., problem gambling, aggressive behavior, risky driving, antisocial behavior) (Kapetanovic et al., 2023; Freund et al., 2022; Te Brinke et al., 2022; X. Zhang et al., 2019; Harris et al., 2015). Given these associations, accurately assessing an individual’s sensation-seeking tendencies is crucial for prevention and intervention efforts.

### 1.1. Related Work

Current mainstream methods fail to accurately measure sensation seeking. Despite their limitations, self-report questionnaires remain the predominant method for measuring sensation seeking. However, their accuracy depends critically on truthful participant response, potentially introducing measurement bias in high-stakes contexts (e.g., psychiatric diagnosis, competitive selection) where individuals may distort responses to serve their self-interest (Boyle & Helmes, 2009). Given the importance of sensation seeking in predicting mental health and behavioral risks, there is a need for alternative assessment methods that reduce bias in sensitive settings.

Advances in digital phenotyping—leveraging sensor technology, passive data collection, and machine learning (ML)—offer promising solutions. Unlike self-reports, biometric behavioral data captured through sensors are less susceptible to response biases and can provide dynamic, context-dependent behavioral insights (Dlima et al., 2022; Onnela, 2020; Taylor et al., 2020). ML is particularly well-suited for modeling complex relationships between behavioral data and psychological constructs, excelling at detecting nonlinear patterns in high-dimensional datasets (Bishop, 2006). For instance, smartphone-derived behavioral patterns have successfully identified personality traits and impulsivity (H. Wen et al., 2021; Stachl et al., 2020), while tablet sensor data have been used to identify internet gaming disorder (Cho et al., 2024). Given that sensation seeking is closely tied to exploratory and risk-taking behaviors, digital behavioral phenotyping presents a viable alternative to traditional questionnaires.

Gait, a rhythmic whole-body movement, reflects the integration of cognitive, emotional, and physiological processes (Adlou et al., 2025; Mason et al., 2025; Montero-Odasso et al., 2012) and may serve as a behavioral indicator of sensation seeking. The neurobiological basis of sensation seeking, characterized by heightened striatal dopaminergic tone and reduced D2-type receptor availability, modulates approach-avoidance behaviors by increasing preference for intense stimuli (Norbury & Husain, 2015; Sapuram et al., 2021), suggesting that individual differences in sensation-seeking traits may be measurable through gait patterns that reflect risk-taking or exploratory motor tendencies.

Although limited research has directly examined gait–sensation seeking associations, empirical evidence suggests links between gait characteristics and related traits (e.g., impulsivity, openness to experience). For example, higher stride length variability and gait velocity correlate with reduced risk-taking tendencies (Klupp et al., 2023), while inertial measurement unit (IMU) data have enabled accurate Openness measurement (83.6% classification accuracy) (S. J. Chen & Lee, 2024). However, further research is needed to determine whether gait can accurately measure sensation seeking. More importantly, distinct personality traits exhibit correspondingly distinct gait patterns (Satchell et al., 2017; Zhao et al., 2024). To our knowledge, no study has universally established identical gait patterns across different personality traits. Consequently, developing a ML-based model specifically targeting sensation seeking through gait feature analysis is warranted.

The present study therefore investigates the validity of using ML models to identify sensation seeking based on gait data, offering a novel, objective, and non-intrusive assessment approach.

### 1.2. Structure of the Paper

The remainder of this paper is organized as follows. Section 2 details the methodology, including participant information, data acquisition procedures, the preprocessing pipeline for gait data (encompassing skeletal keypoint extraction, coordinate translation, and Gaussian filtering), feature extraction, and the machine learning framework. Section 3 presents the experimental results, covering the general analysis, feature selection outcomes, and the performance comparison of the trained models. Section 4 discusses the implications of our findings, interprets the biomechanical correlates of the identified gait features, acknowledges the study’s limitations, and suggests directions for future research. Finally, Section 5 concludes the paper by summarizing the main contributions and outlining potential applications.

## 2. Methods

This study received ethical approval from the Institutional Review Board at the Institute of Psychology, Chinese Academy of Sciences, China’s sole national-level comprehensive research institution in psychology (Protocol No. H18022; approval period: October 2018–December 2020). Informed consent was obtained from all participants involved in the study.

The research process comprised three phases: (a) data collection, (b) data preprocessing, and (c) data modeling.

### 2.1. Data Collection

The participants comprised 233 healthy Chinese adults (gender: 106 males, 127 females, approximately 1:1.2; age: 22.72 ± 2.58 years). All participants were postgraduate students. Exclusion criteria were: self-reported history of neurological disorders (e.g., stroke, Parkinson’s), musculoskeletal conditions significantly affecting gait (e.g., recent lower limb injury, severe arthritis), or current psychiatric diagnosis.

**Figure 1:**
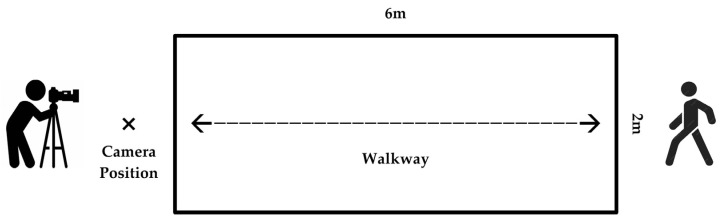
Schematic representation of the experimental setup.

Participants were required to perform multiple round-trip walking trials along a 6 m × 2 m straight walkway at their natural pace, with each session lasting 2 min. This was a deliberate choice to maximize ecological validity, as gait patterns at a preferred speed may be more representative of an individual’s natural walking biomechanics and are less constrained than fixed-speed paradigms. Gait was recorded using a high-resolution Sony camera (25 fps; Sony, Tokyo, Japan) positioned to capture frontal and posterior views (Figure 1). Environmental conditions (lighting, walkway surface) were rigorously controlled.

Following the walking trials, participants were instructed to complete the 8-item Brief Sensation-Seeking Scale for Chinese (BSSS-C) (X. Chen et al., 2013), rating each item on a 5-point Likert scale (1 = strongly disagree to 5 = strongly agree). Higher aggregate scores on the BSSS-C reflected stronger sensation-seeking tendencies, with this validated metric serving as the gold-standard reference for ML feature selection and model training.

### 2.2. Data Preprocessing

#### 2.2.1. Data Unification

Following data acquisition, all gait videos underwent preprocessing to remove artifacts and ensure temporal consistency.

First, the gait data were processed with frontal-view prioritization. The current dataset consisted of multi-view gait recordings, including frontal-view and posterior-view sequences. According to previous studies, frontal views may capture more discriminative features than posterior views (Y. Wen et al., 2022; Fang et al., 2019). Thus, for each participant, their frontal-view gait sequences were preferentially selected and processed.

Second, all unobstructed frontal-view gait sequences were cropped to a uniform length of 50 consecutive frames (approximately 2 s) per participant to ensure at least one full gait cycle was captured.

#### 2.2.2. Skeletal Keypoints Extraction

**Figure 2:**
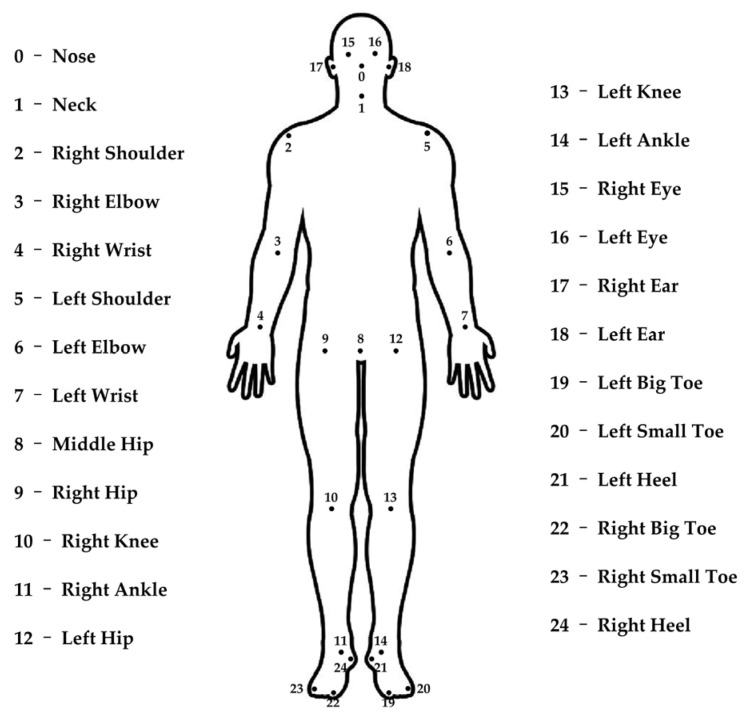
25 skeletal keypoints.

To characterize spatiotemporal variations in human gait patterns, precise detection of skeletal keypoints (e.g., hip, knee, and ankle joints) and continuous tracking of their positional dynamics are essential. OpenPose, a computer vision framework for human pose estimation (Cao et al., 2021), facilitates this process by identifying anatomical keypoints and algorithmically constructing a standardized skeletal model from spatial coordinates, enabling quantitative gait analysis. In this study, the OpenPose system (v1.7.0) was employed to automatically extract 2D coordinate data (x, y) for 25 keypoints per image frame for each participant (see Figure 2).

#### 2.2.3. Coordinate Translation

When using OpenPose for skeletal keypoint estimation, the extracted coordinates are initially referenced to the global image coordinate system, where the origin is conventionally positioned at the top-left corner of the frame. Direct application of these raw coordinates in subsequent analyses may introduce systematic biases, potentially distorting biomechanical or behavioral inferences. Such biases primarily stem from the dependence of unprocessed coordinates on extrinsic factors (e.g., camera placement, subject-to-camera distance) rather than intrinsic movement patterns.

**Figure 3:**
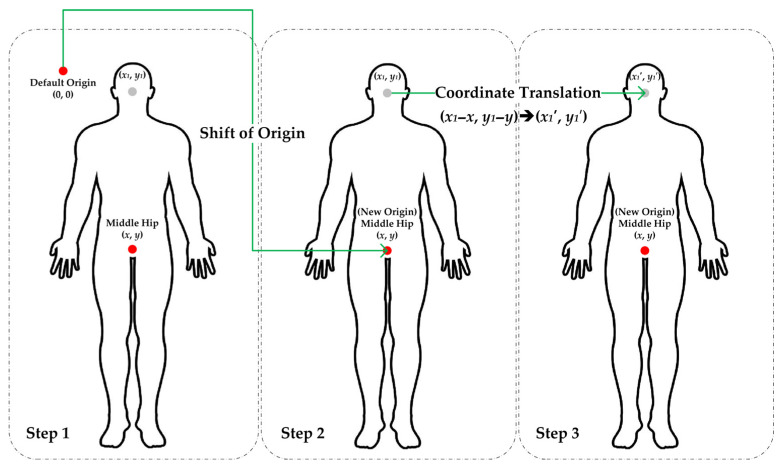
Coordinate translation.

To address these limitations, coordinate translation serves as a critical preprocessing step to enhance data robustness, interpretability, and cross-study comparability. In this study, raw coordinates (referenced to image space) were transformed to a hip-centered coordinate system (the middle hip joint redefined as the new origin) to remove camera-dependent biases (Figure 3):

(*xi*′, *yi*′) = (*x_i_* − *x*_Middle Hip_, *y_i_* − *y*_Middle Hip_) (1)

where (*x*_Middle Hip_, *y*_Middle Hip_) represents the mid-hip joint position.

#### 2.2.4. Noise Removal

Vision-based pose estimation systems, such as OpenPose, are susceptible to high-frequency artifacts arising from both sensor limitations (e.g., pixel quantization, variable illumination) and algorithmic jitter in joint localization. These perturbations generate temporally discontinuous joint trajectories that contravene the smoothness constraints inherent to human locomotion, which is governed by musculoskeletal dynamics. To address these noise sources while maintaining biomechanically valid motion patterns, Gaussian filtering was applied to denoise skeletal keypoint data obtained during human walking.

The Gaussian filter—a prevalent low-pass operator in image processing—functions by attenuating high-frequency spectral components while preserving signal integrity. In this study, this linear smoothing technique was implemented via discrete convolution of the input signal with a Gaussian kernel. This operation assigns weighted coefficients that decay exponentially with spatial distance, prioritizing proximal data points while progressively diminishing the influence of distal elements. The resultant spatial filtering produces an edge-preserving smoothing effect that achieves significant suppression of high-frequency noise artifacts and retention of critical structural boundaries. Furthermore, Gaussian filtering exhibits high computational efficiency, facilitates straightforward implementation in embedded/real-time systems, and features easily tunable parameters.

In this study, the convolution kernel of the Gaussian filter was c = [1, 4, 6, 4, 1]/16, and its formula was as follows:

*out*[*i*] = 1/16(*in*[*i*] × 1 + *in*[*i* + 1] × 4 + *in*[*i* + 2] × 6 + *in*[*i* + 3] × 4 + *in*[*i* + 4] × 1)

This processing approach ensures that the denoised trajectories maintain physiologically plausible derivatives (velocity and acceleration profiles), which are fundamental for deriving accurate biomechanical metrics, including stride kinematics and inter-joint coordination patterns.

#### 2.2.5. Feature Extraction

Following comprehensive preprocessing, from the preprocessed data, we extracted a total of 300 gait features per participant.

Specifically, each participant’s gait data was transformed into time-series representations consisting of 50 consecutive frames. Each frame contained 2D spatial coordinates (x, y) for 25 skeletal keypoints, yielding 50 time-series sequences per participant (25 keypoints × 2 coordinates = 50 time-series sequences). Six distinct time-series features were then extracted from each sequence (Table 1), resulting in a total feature space of 300 gait parameters per participant (50 sequences × 6 time-series features = 300 gait features). It is worth noting that, due to the scarcity of research directly investigating the relationship between gait characteristics and sensation seeking (and its related traits), there is currently no established framework for extracting relevant time-series features. However, a previous study indicates an association between gait variability and sensation seeking-related behaviors (e.g., risk-taking) (Klupp et al., 2023). Consequently, in addition to the commonly used statistical indicators for quantifying central tendency (F1 mean), dispersion (F2 standard deviation), and data distribution (F3 skewness, F4 kurtosis), we extracted two time-series features to quantify global fluctuation amplitude (F5 sum of squared coordinate values) and local fluctuation amplitude (F6 cumulative sum of absolute differences between adjacent data points). Finally, all 300 extracted gait features were z-score standardized prior to analysis.

The adopted feature naming convention follows a hierarchical structure: keypoint number (0–24, see Figure 2), coordinate (x/y), and time-series feature (F1–F6, see Table 1). For example, J0_X_F1 denotes the mean value (F1) of the x-coordinate (X) time-series for the nose joint (J0); J1_Y_F2 represents the standard deviation (F2) of the y-coordinate (Y) time-series for the neck joint (J1).

### 2.3. Data Modeling

Following data pre-processing, ML models were developed using the Waikato Environment for Knowledge Analysis (WEKA, version 3.8.6) to assess BSSS-C scores from gait features.

In this study, a nested cross-validation procedure was implemented in Weka to ensure a rigorous and unbiased evaluation of the predictive model. The analysis utilized the AttributeSelectedClassifier meta-classifier. Feature selection was performed using the correlation-based feature subset evaluator (CfsSubsetEval) in conjunction with the GreedyStepwise search method. This filter-based approach identifies a feature subset that maximizes predictive correlation with the target variable while minimizing inter-feature redundancy. The selected features were then used to train an AutoWEKAClassifier, which performs automated algorithm selection, hyperparameter tuning, and internal 10-fold cross-validation. The entire pipeline was evaluated using an outer 10-fold cross-validation scheme.

Specifically, the outer 10-fold cross-validation framework guarantees that the feature selection and model training processes at each fold are conducted on an independent partition of the data, with a held-out test set used solely for final evaluation. This prevents any data leakage and provides an unbiased estimate of the model’s true generalization performance on unseen data. Furthermore, the nested procedure evaluates the entire modeling pipeline—feature selection and model optimization—as a single integrated process. The reported performance thus reflects the expected efficacy of the complete methodology when deployed on new data, enhancing the reproducibility and practical validity of the results. The use of CfsSubsetEval further strengthens this process by providing a computationally efficient and generalizable feature selection step based on stable statistical properties rather than a specific algorithm.

Modeling performance was evaluated by four metrics, including Correlation Coefficient (CC), Mean Absolute Error (MAE), Root Mean Squared Error (RMSE), and R^2^. The CC measures the alignment of model outputs with observed values through linear mapping relationships. The MAE quantifies the average deviation magnitude, while the RMSE evaluates error magnitude through the square root of averaged squared residuals, emphasizing larger errors. The R^2^ assesses the proportion of variance in the observed data that is explained by the model.

## 3. Results

### 3.1. General Analysis

The study cohort comprised 233 participants with BSSS-C scores ranging from 11 to 38 (24.22 ± 5.35). This distribution demonstrated a small effect size (Cohen’s *d* = 0.13) when compared to the original validation sample (25.77 ± 6.67) from X. Chen et al. (2013). As presented in Table 2, multiple gait features showed statistically significant correlations with BSSS-C scores.

### 3.2. Feature Selection

Using the CfsSubsetEval feature selection algorithm—which prioritizes feature subsets’ collective explanatory power for mapping relationships—we identified an optimal combination of 18 gait parameters that maximized model performance, distinct from individual features’ correlational strengths. Table 3 presents the standardized *β* coefficients (*β*) quantifying the linear associations between these selected gait features and BSSS-C scores, and indicates no evidence of severe multicollinearity among the selected gait characteristics, with all tolerance values exceeding 0.10 and all VIF values below 10.

### 3.3. Model Training

Three distinct ML models (SMO Regression, Multilayer Perceptron, and Bagging) were trained, respectively. As detailed in Table 4, the SMO Regression model demonstrated superior performance (*r* = 0.60, MAE = 3.50, RMSE = 4.59, R^2^ = 0.26), outperforming the other approaches.

## 4. Discussion

This study establishes that gait kinematics serve as promising behavioral indicators for measuring sensation-seeking traits, successfully extending biomechanical analysis into the domain of psychological assessment.

First, gait characteristics can be recognized as potential indicators for measuring sensation-seeking characteristics. Emerging evidence demonstrates that gait patterns-as a manifestation of neuromotor control-exhibit systematic relationships with psychological constructs across cognitive, affective, and personality domains (Avanzino et al., 2018; S. J. Chen & Lee, 2024; Satchell et al., 2017). Our findings extend this paradigm by showing that gait patterns capture meaningful variance in sensation-seeking traits. In the present study, based on gait features, the sensation-seeking identification model performed well according to the metrics used to assess the performance of ML models. Specifically, the observed metrics (CC = 0.60 [moderate correlation]; MAE = 3.50; RMSE = 4.59; R^2^ = 0.26) fell within the range reported in comparable psychological and psychiatric studies (CC: 0.47–0.73; MAE: 3.3–8.9; RMSE: 4.55–11.57; R^2^: 0.11–0.41) (Y. Zhang et al., 2025; Crocamo et al., 2025; Weisenburger et al., 2024; Y. Wen et al., 2022). Moreover, the small effect size (Cohen’s *d* = 0.13) in BSSS-C scores between this cohort and the original validation sample (X. Chen et al., 2013) tentatively supports sample representativeness within this demographic context. However, it is worth noting that the model’s current performance—positioned as a proof of concept rather than a validated clinical tool—underscores the need for enhanced accuracy and validation in larger, more diverse cohorts.

Second, the ML method facilitates the identification of biomechanical markers associated with sensation-seeking traits. Feature selection yielded 18 gait parameters demonstrating promising associations with BSSS-C scores. The ranking of feature importance (such as SHAP values or permutation importance) was not employed in this study due to our primary focus on establishing overall predictive validity and the fact that our chosen feature selection method (CfsSubsetEval) prioritized subset predictive power over individual feature rankings. Furthermore, our best-performing model (SMO Regression) operates in a high-dimensional kernel space, making global feature importance metrics less straightforward to interpret. Therefore, we instead prioritized reporting the linear associations (standardized β coefficients) for selected feature subset (see Table 3). These associations were interpreted within established frameworks linking gait kinematics to psychological states. Sensation seeking has a neurobiological basis that heightens preferences for intense stimuli to modulate approach-avoidance behaviors (e.g., risk-taking) (Norbury & Husain, 2015; Sapuram et al., 2021). Prior research indicates that heightened sensation seeking correlates with diminished cognitive control during emotion regulation (Deng & Zhang, 2020; J. Li et al., 2023), which may subsequently exacerbate emotional dysregulation, anger, and risky behaviors (Bogdan et al., 2016; Sârbescu & Rusu, 2021). In this study, pronounced skewness (F3) values at neck (J1_Y_F3: *β* = −0.12), elbow (J6_Y_F3: *β* = 0.10), shoulder (J5_X_F3: *β* = 0.09) and wrist (J4_Y_F3: *β* = −0.04) suggest asymmetric joint kinematics, potentially reflecting biases towards flexion or extension. Such postural asymmetry may increase postural sway magnitude and frequency, which has been linked to emotion dysregulation (e.g., instability, mood disorder) and high-arousal states like anger (Elboim-Gabyzon et al., 2022; Deligianni et al., 2019; Pieruccini-Faria et al., 2018). Additionally, the negative association for ankle kurtosis (J14_X_F4: *β* = −0.15) may reflect increased smoothness in joint motion trajectories, a pattern tentatively associated with anger in prior work (Kang & Gross, 2016). We emphasize that these biomechanical-emotional interpretations remain hypothetical and warrant direct empirical validation through concurrent emotion-gait assessment. Elucidating relationships between gait features and sensation seeking could refine behavioral phenotyping frameworks for high-sensation seekers. Moreover, we also acknowledge that advanced model interpretation frameworks, such as SHAP, could provide deeper, local insights into how specific gait patterns contribute to individual-level predictions of sensation seeking. Their application will be particularly valuable in future studies with larger and more diverse datasets, where such techniques can help unravel complex, nonlinear feature interactions and enhance the personalization and explanatory power of digital phenotyping models.

Third, the use of gait analysis and ML methods facilitates objective, real-time, non-invasive identification of sensation seeking in non-laboratory scenarios. The integration of gait analysis and ML methodologies represents a paradigm shift in behavioral measurement, enabling objective and ecologically valid assessment of sensation seeking beyond traditional laboratory constraints. This approach circumvents longstanding limitations of self-report instruments (e.g., recall bias, social desirability effects) by quantifying subtle movement patterns through wearable sensors or vision-based systems, while ML algorithms decode these kinematic signatures into psychometrically meaningful constructs. The real-time processing capacity of embedded ML models facilitates automatic monitoring of sensation seeking during daily activities, opening up the possibility of personalized and timely psychological and educational interventions. From a methodological perspective, this technological synergy addresses critical gaps in behavioral science research by standardizing measurement scales across diverse populations and settings, while preserving the ecological validity inherent to non-laboratory observation. The non-invasive nature of automated gait analysis further removes barriers to large-scale implementation in educational and community settings, permitting longitudinal tracking of behavioral trajectories without disrupting natural routines. This advancement not only expands research possibilities in developmental psychopathology and educational neuroscience but also establishes a scalable framework for early identification of risk phenotypes across the mental health continuum. Future iterations incorporating multimodal biometric integration could further refine the validity of this approach, ultimately bridging the persistent gap between laboratory-derived constructs and real-world behavioral manifestations.

Fourth, algorithms need to be tailored for biosignal processing. In this study, different algorithms vary in their modeling performance when dealing with the same ML task. Specifically, the SMO Regression optimized via the Sequential Minimal Optimization (SMO) algorithm, demonstrated superior performance in measuring sensation seeking through gait feature analysis compared to Multilayer Perceptron and Bagging. SMO Regression derives its generalization strength from the principle of structural risk minimization, which systematically balances model complexity (controlled by minimizing the norm of weights) and empirical error. Unlike classification-oriented SVMs that maximize separation margins, SMO Regression employs an ε-insensitive loss function to tolerate small deviations while enforcing model flatness in feature space. This mechanism reduces overfitting risks, particularly in high-dimensional or limited-sample scenarios (e.g., 233 participants and 300 features). The integration of slack variables further enhances robustness by accommodating outliers and measurement noise. In contrast, Multilayer Perceptron is inherently susceptible to overfitting under small-sample conditions. Their sensitivity to input perturbations may also amplify noise in gait data. While ensemble methods such as Bagging improve stability via variance reduction, their efficacy in high-dimensional sparse datasets can diminish when irrelevant features dominate, as recursive feature selection is not inherently prioritized in standard implementations. Additionally, bagging’s reliance on majority voting or averaging offers less explicit control over outlier sensitivity compared to SMO Regression’s slack variable framework. The kernelized formulation of SMO Regression further enables flexible modeling of nonlinear relationships without sacrificing robustness, a critical advantage for capturing complex associations between gait and sensation seeking.

Despite promising results, this study has limitations. First, the sample size (N = 233) and restricted demographic diversity (young, educated Chinese adults) limit the immediate generalizability of our findings. Furthermore, while our preprocessing pipeline focused on relative kinematics, the potential influence of anthropometric factors such as body mass index (BMI) was not explicitly controlled for. Although the cohort’s homogeneity (healthy young adults) inherently limits extreme BMI variations, and our hip-centered coordinate preprocessing (Section 2.2.3) mitigates biases related to absolute body size by focusing on relative kinematics, future validation across more diverse populations with collected anthropometric data is still necessary to confirm the generalizability of the findings. Most importantly, the model requires external validation on an independent and more diverse sample to confirm its generalizability and translational potential beyond the current dataset. Second, emerging computational techniques (e.g., graph neural networks) may better model complex spatiotemporal dependencies than the algorithms tested here. Moreover, future research should integrate diverse gait analysis methods—such as frequency-domain techniques for rhythmic dynamics and continuous joint-angle kinematics for inter-segmental coordination—to enrich biomechanical phenotyping, enhance assistive device personalization, and uncover novel signatures of locomotor behavior in both clinical and robotic applications (Salomon et al., 2024; M. Li et al., 2022). Third, while kinematic features showed predictive validity, integrating physiological signals (e.g., EEG, heart rate variability) could elucidate sensation seeking’s neurobehavioral mechanisms more holistically. Fourth, the correlational design precludes causal inference; longitudinal studies are needed to determine directional relationships between gait patterns and trait development. Finally, the current investigation was restricted to sensation seeking, thereby limiting cross-trait comparisons of gait-personality relationships. Subsequent studies should employ multivariate approaches to dissociate gait features that are shared across personality dimensions from those that are uniquely associated with specific traits.

## 5. Conclusions

This study pioneers the use of gait analysis and ML to quantify sensation-seeking traits, demonstrating that kinematic traits can be used to measure psychological dimensions accurately. The success of SMO Regression highlights the value of tailored algorithms for biomedical signal processing. Future work should expand datasets, integrate multimodal sensors, and explore causal pathways to translate these findings into clinical or behavioral interventions. By bridging motor control and psychological traits, this research opens new avenues for objective, real time and non-invasive psychological assessment.

## Figures and Tables

**Table 1:** Time-series features extracted from each time-series sequence.

Features	Description
F1	Mean coordinate value
F2	Standard deviation of coordinates
F3	Distribution skewness
F4	Distribution kurtosis
F5	Sum of squared coordinate values
F6	Cumulative sum of absolute differences between consecutive observations

**Table 2:** Correlations between gait features and BSSS-C scores (N = 233).

Statistically Significant Features	*r*
J0_X_F2	−0.16 *
J1_X_F2	−0.15 *
J2_X_F2	−0.14 *
J2_Y_F4	0.16 *
J5_X_F2	−0.17 **
J8_X_F2	−0.17 **
J10_X_F2	−0.16 *
J12_X_F2	−0.15 *
J13_X_F2	−0.14 *
J15_X_F2	−0.14 *
J16_X_F2	−0.17 *
J17_Y_F6	−0.16 *
J18_X_F2	−0.17 **
J20_X_F6	0.14 *
J24_X_F2	−0.13 *

Note. * *p* < 0.05, ** *p* < 0.01. The adopted feature naming convention follows a hierarchical structure: keypoint-number (0–24)_coordinate (x/y)_time-series-feature (F1–F6). Only statistically significant features are listed here (*p* < 0.05).

**Table 3:** Standardized *β* coefficients and collinearity diagnosis of selected gait features (N = 233).

Selected Gait Features	Standardized *β*	Tolerance	VIF
J1_Y_F3	−0.12	0.92	1.09
J2_X_F2	−0.09	0.49	2.06
J2_X_F4	0.11	0.88	1.13
J2_Y_F4	0.09	0.90	1.12
J4_Y_F3	−0.04	0.91	1.10
J5_X_F2	−0.12	0.18	5.51
J5_X_F3	0.09	0.79	1.27
J6_Y_F3	0.10	0.74	1.35
J6_Y_F4	0.05	0.72	1.38
J10_X_F6	−0.08	0.81	1.23
J12_X_F6	−0.05	0.73	1.38
J14_X_F4	−0.15	0.91	1.09
J17_Y_F4	0.06	0.82	1.22
J17_Y_F6	−0.07	0.80	1.25
J18_X_F2	−0.06	0.18	5.69
J19_X_F3	−0.11	0.92	1.09
J20_X_F6	0.21	0.84	1.19
J22_X_F4	−0.08	0.92	1.09

Note. The adopted feature naming convention follows a hierarchical structure: keypoint–number (0–24)–coordinate (x/y)–time-series feature (F1–F6).

**Table 4:** Performance of ML models.

ML Models	CC (*r*)	MAE	RMSE	R^2^
SMO Regression	0.60	3.50	4.59	0.26
Multilayer Perceptron	0.46	3.73	4.73	0.22
Bagging	0.53	3.51	4.58	0.26

## Data Availability

The data presented in this study are available on request from the corresponding author due to ethical reasons.
